# Phylogenetic analysis reveals two genotypes of the emerging fungus *Mucor indicus,* an opportunistic human pathogen in immunocompromised patients

**DOI:** 10.1038/emi.2017.51

**Published:** 2017-07-12

**Authors:** Saad J Taj-Aldeen, Muna Almaslamani, Bart Theelen, Teun Boekhout

**Affiliations:** 1 Mycology Unit, Microbiology Division, Department of Laboratory Medicine and Pathology, Hamad Medical Corporation, PO Box 3050, Doha, Qatar; 2 Weill Cornell Medicine-Qatar, Education City, PO Box 24144, Doha, Qatar; 3 Infectious Disease Division, Department of Medicine, Hamad Medical Corporation, PO Box 3050, Doha, Qatar; 4 Westerdijk Fungal Biodiversity Institute (formerly CBS Fungal Biodiversity Centre), 3584 CT, Utrecht, The Netherlands; 5 Institute of Biodiversity and Ecosystem Dynamics (IBED), University of Amsterdam, PO Box 19268, Amsterdam, The Netherlands

**Keywords:** disseminated infection, genotypes, liposomal amphotericin B, liver transplant, mucormycosis, *Mucor indicus*

## Abstract

Mucormycosis is a rare fungal infection caused by *Mucor indicus*. Phylogenetic analysis of many *M. indicus* isolates, mainly sampled from different clinical and environmental specimens collected worldwide, revealed two genotypes, I and II, based on ITS and D1/D2 LSU rDNA sequences. A retrospective review of the literature revealed 13 cases. Eight (76.9%) patients had disseminated infections, and the overall mortality rate was 30.7%. A pulmonary infection caused by *M. indicus* genotype I in a liver transplant recipient was disseminated to include the skin and was successfully treated with liposomal amphotericin B and aggressive surgery. *M. indicus* can infect a wide variety of patients with no real preference for the site of infection. We concluded that *M. indicus* has emerged as a significant cause of invasive mycosis in severely immunocompromised patients worldwide. Early diagnosis and initiation of appropriate therapy could enhance survival in these immunocompromised patient populations.

## INTRODUCTION

Mucormycosis is a fungal infection caused by a variety of genera belonging to the order Mucorales. The disease has re-emerged as a cause of significant, life-threatening infections, particularly in immunocompromised patients.^1^ The infection is most often acquired by inhalation of spores from environmental sources, by direct inoculation due to skin lesions or rarely by ingestion of contaminated food.^2^ The disease caused by Mucormycetes is characterized by angioinvasion leading to thrombosis, infarction and necrosis of the involved tissue.^3^ The common clinical manifestations include rhinocerebral, cutaneous, pulmonary, gastrointestinal and disseminated infections. In solid-organ transplant recipients, the disease occurs at a rate of two cases per 1000 transplant surgeries^4^ the majority of these cases occur in renal transplant patients, with a mortality rate of 38% to 56.5%.^5^ The most common manifestation of the disease is rhino-orbito-cerebral, but pulmonary and cutaneous infections occur to a lesser extent.^4, 6^ Disseminated mucormycosis is a very rare and life-threatening form of the disease.^7, 8^ The prognosis of disseminated mucormycosis is poor, with reported mortality rates in the neutropenic population ranging from 46% to 100%.^9, 10^ In this paper, we describe the existence of two genotypes, I and II, of the emerging pathogenic fungus *Mucor indicus* and a case of disseminated mucormycosis in a liver transplant patient caused by genotype I. The infection was successfully treated with liposomal amphotericin B in combination with surgical intervention.

## MATERIALS AND METHODS

### Strains

Two clinical strains of *M. indicus, viz.,* Q1088 and Q1106, isolated from a liver transplant patient at Hamad Hospital, Doha, Qatar, were studied and characterized during this study. In addition, we also analyzed the sequences of three isolates from clinical sources obtained from Dr Josepa Gené at Unitat de Microbiologia, Facultat de Medicina I Ciències de la Salut, Universitate Rovira I Virgilli, Reus, Spain and 24 reference strains from Westerdijk Fungal Biodiversity Institute (formerly CBS Fungal Biodiversity Centre, Table 1). The clinical strains of *M. indicus* were characterized for their genotypes during this study.

### Isolation

Tissue, bronchoalveolar lavage (BAL) and bronchial wash (BW) specimens from the patient were processed for direct microscopy and cultured as previously described for filamentous fungi.^11^ Four drops each of BAL and BW were used to inoculate culture plates, while small pieces of tissue were inoculated into brain heart infusion broth and then onto two sets of plates containing Sabouraud dextrose agar (Oxoid Ltd., UK) +40 U/mL streptomycin and 20 U/mL penicillin (SDA+SP), Sabouraud dextrose agar (without antibiotics) (SDA), and brain heart infusion agar (Mast Diagnostics, UK)+40 U/mL streptomycin and 20 U/mL penicillin. Each plate was inoculated with four drops; one set of plates was incubated at room temperature and the other at 37 °C. Fungal growth appeared on all types of media within 48 h as fluffy golden gray cottony colonies. The fungus grew faster at 37 °C but was able to grow at 40 °C. BAL or tissue samples were mounted in 30% KOH and visualized under a light microscope at × 400 magnification. Ribbon-like, broad and non-septate branching hyphae were evident in this preparation.

### Fungal examination

A wet preparation from colonies revealed broad, non-septate branching hyphae and sporangia. The sporangia were globose, yellow to brown and with a spherical columella. Sporangiospores were smooth walled, subspherical to ellipsoid and consistent with those of fungi of the order Mucorales. In contrast to most other *Mucor* species, *M. indicus* is thermotolerant to temperatures up to 40 °C.^12^ Based on the cultural and microscopic characteristics, the strains isolated from all specimens were tentatively identified as *M. indicus*. To visualize fungal hyphae in the tissue, part of the tissue specimen was embedded in paraffin and used to prepare histological slides. A combination of histological stains, hematoxylin and eosin and Gomori methenamine silver, were used to visualize both the tissue reaction and the fungal etiology.

### Molecular identification

Strains were maintained on malt extract agar for 48 h at 25 °C. Genomic DNA was extracted according to Bolano *et al.*^13^ with minor alterations. The internal transcribed spacers (*ITS1* and *ITS2*, including the 5.8S gene) and the D1/D2 region of the large subunit ribosomal DNA (*LSU rDNA*) were amplified and sequenced per methods described previously by Cendejas-Bueno *et al.*^14^ The sequences were trimmed and assembled, and consensus sequences were created with SeqMan Pro software (Version 9.0.4, 418, DNAStar Inc., Madison, WI, USA). The obtained sequences were compared with the available data in the NCBI database using the Basic Local Alignment Search Tool (BLAST).^15^ Sequences of the studied strains and downloaded reference sequences from the BLAST search were aligned with MegAlign (DNAStar) using the Clustal W method.

### Phylogeny

Phylogenetic trees were created from the aligned sequences with MEGA6^16^ using maximum likelihood based on the Hasegawa-Kishino-Yano model^17^ and by applying 1000 bootstrap repetitions.

## RESULTS

### Case study

**Figure 1:**
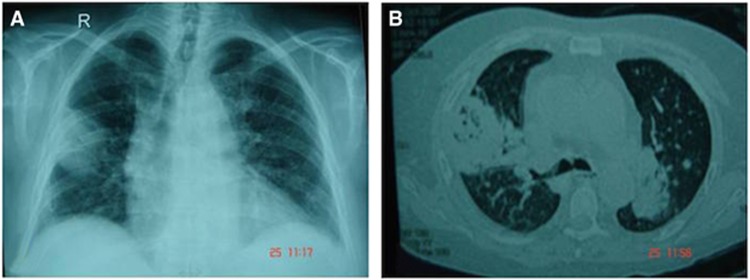
Imaging of the chest. (**A**) X-ray showing the thick-walled cavity (fungal mass) at the base of the right upper lobe. (**B**) A high-resolution CT scan shows bilateral opacities.

A 55-year-old Qatari male underwent orthotopic liver transplantation for progressive liver dysfunction due to alcoholic liver cirrhosis in January 2007. His medical history included hypertension and alcohol consumption. The immunosuppressive therapy consisted of tacrolimus and mycophenolate mofetil. The clinical course was uncomplicated until 5 months after the operation, when he developed a *Pneumocystis jiroveci* infection with bronchiolitis obliterans organizing pneumonia and portal vein thrombosis, which were treated with IV cotrimoxazole and high-dose methyl prednisolone 60 ml Q8 h. He was discharged on cotrimoxazole 800/160 mg every other day and prednisolone 60 mg orally. At admission on 20 October 2007, the patient presented with a history of fever (38.5 °C), productive cough of 1 day duration, history of diarrhea of 3 days duration and bilateral leg pain. At the time, the immunosuppressants tacrolimus (trough serum levels of 10 ng/mL) and prednisone 35 mg daily were administered. Physical examination on admission was normal. Laboratory tests revealed WBC 1500/mm^3^, absolute neutrophils 400/mm^3^, hemoglobin 82 mg/mL, platelets 85 000/mm^3^, and glucose 17.4 mmol/L. Liver and kidney functions were normal. Routine stool microscopy and culture, and the *Clostridium difficile* toxin assay were negative. A Doppler study of the deep venous system revealed bilateral deep vein thrombosis. As the patient was diagnosed with diabetes mellitus, acute deep vein thrombosis and pancytopenia secondary to drugs, cotrimoxazole and mycophenolate mofetil were stopped. The patient discharged himself against medical advice. He was readmitted 3 days later with a history of fever. Laboratory tests revealed WBC 14700/mm^3^. Chest radiography showed a thick-walled cavity at the base of the right upper lobe (Figure 1A). A high-resolution computer tomography (CT) scan showed bilateral opacities with air bronchograms occupying the upper, posterior and peripheral parts of the lungs (Figure 1B). BAL and BW samples obtained from a bronchoscopy were sent to the microbiology laboratory, and cultures revealed the growth of a Mucormycete that was later identified as *M. indicus.*

**Figure 2:**
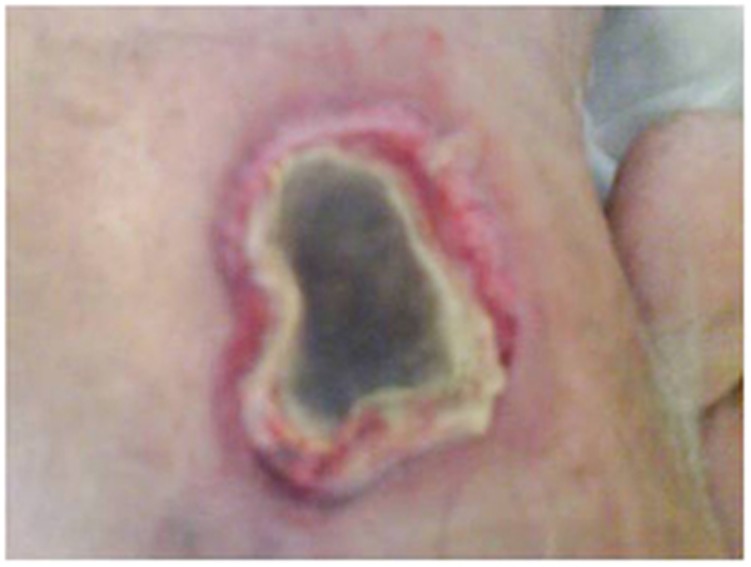
Necrotic ulcer of the skin above the knee with excessive redness and swelling around the wound.

**Figure 3:**
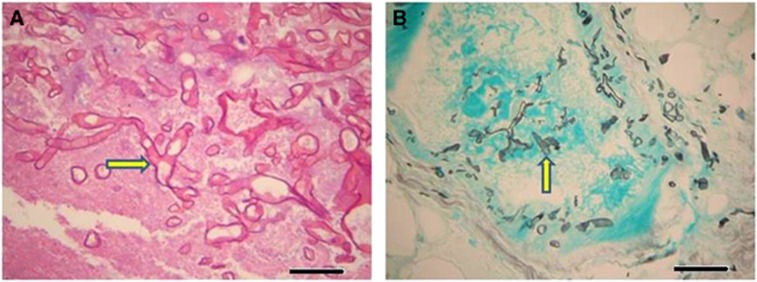
Dissemination of fungal hyphae of *M. indicus* into the tissue of the skin ulcer showed ribbon-like, right-angle branching hyphae. (**A**) Fungal hyphae (arrow) in skin tissue stained with hematoxylin and eosin (× 400), bar=10 μm. (**B**) Darkly stained right-angle branched hyphae of *M. indicus* (arrow) on a green-stained cellular background as seen in histological sections of the skin tissue of the ulcer showed blood vessel invasion. Stained with Gomori methenamine silver (× 200), bar=20 μm.

The antifungal susceptibility minimum inhibitory concentration (μg/mL) profile was as follows: amphotericin B 0.03, itraconazole >8, voriconazole >8, posaconazole 2 and caspofungin 8. The patient started on piperacillin/tazobactam and liposomal amphotericin B 5 mg/kg/day (250 mg IV QD), but he absconded after two days. The patient was readmitted 5 days later with a history of high-grade fever (38.3 °C) and skin lesions. Physical examination showed no abnormalities except that the patient had developed two necrotic skin lesions with pus, one below the left knee over the left shaft measuring 3 cm × 3 cm, and one on the left thigh measuring 6 cm × 3 cm, both with sloping edges and a painful pus base covered with necrotic tissue (Figure 2). CT scans of the head and sinus were normal. Medication was resumed with liposomal amphotericin B 5 mg/kg/day and meropenem 1 mg IV Q8 h. The skin eschars were debrided, and cultures derived from the tissue revealed the growth of *M. indicus*, *Escherichia coli* and *Pseudomonas aeruginosa*. Histopathological examination confirmed the presence of necrotic soft tissue characterized by a sheet of neutrophils and Mucormycetes hyphae (Figure 3). After 3 weeks of antifungal therapy, a new specimen from the skin lesions was negative for bacterial and fungal growth. A repeated CT scan of the chest showed a nodular cavitation lesion with thick walls located peripherally in the posterior segment of the right upper lobe without any calcification. A right anterolateral 5th intercostal thoracotomy with the removal of the fungal ball was done after two months of antifungal therapy. Gram staining and culture of the tissue showed fungal hyphae, which failed to grow. A histopathological lung biopsy was consistent with Mucormycetes hyphae. The patient was discharged in good condition after he received antifungal therapy for 3 months and surgical intervention.

### Phylogenetic analysis

**Figure 4:**
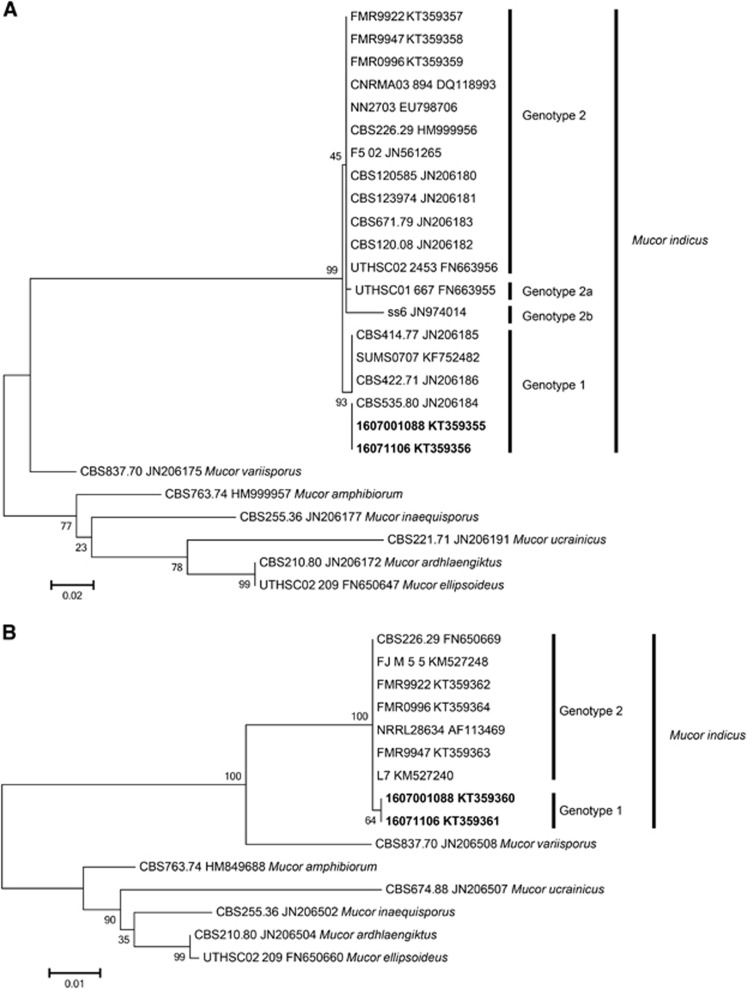
Phylogenetic ML tree with 1000 bootstrap replications. (**A**) Based on the *ITS1*, *5.8S*, and *ITS2* regions of the ribosomal DNA. The scale bar indicates 0.02 substitutions per site. (**B**) Based on the *D1/D2* region of the large subunit of the ribosomal DNA. The scale bar indicates 0.01 substitutions per site. Accession numbers in bold are sequences created in this study.

The morphological traits of fungi can vary substantially from culture to culture and are subject to environmental influences.^18^ Thus, phenotypic characteristics may be subject to ambiguities induced by environmental stress. However, recent molecular techniques have been introduced to provide more objective criteria for fungal identification. To explore the phylogenetic relationships among the clinical isolates and global reference isolates, we analyzed the nucleotide sequences of the two new isolates in the present study and three clinical isolates from invasive infections; FMR 9922 was obtained from peritoneal fluid and the other two isolates (FMR 9947, and FMR 9996) were from liver abscesses (Table 1). These strains were analyzed and aligned with data from 19 clinical and environmental reference strains. Unexpectedly, the sequences of the *M. indicus* isolates investigated in our study were classified into two distinct groups in the phylogenetic tree based on the *ITS1+2* and *LSU rDNA* sequences (Figure 4). Group 1 (designated herein as genotype 1) included seven strains, viz., the two strains from Qatar, Q1088 and Q1106, with four reference strains from CBS, including three environmental strains, CBS 414.77 from India, CBS 422.71 from Indonesia, and CBS 535.80 from South Africa, and one clinical strain, SUMS0707, from China (Table 1). In contrast, the FMR strains were grouped in genotype 2 together with 15 clinical and environmental strains isolated from all over the world (Table 1). Substantial genetic diversity was observed. There were four mutations in the *ITS* sequences that distinguished the two *M. indicus* genotypic groups. Three of these were single nucleotide substitutions (C−T, A−T, G−A), and one was an insertion/deletion. Furthermore, there were two sequences from GenBank that showed some deviations from the rest of the sequences. The sequence with accession number JN974014 showed six mutations (4 × A>T, 1 × C>A, 1 × G>A) compared to the other sequences in genotype 2, and the sequence with accession number FN663955 exhibited a single nucleotide substitution at two positions (A>G and T>G). Two strains, ss6 and UTHSCO1667, showed some unique nucleotide substitutions in the *ITS1*, *5.8S* and *ITS2* regions that placed them apart from both genotype 1 and 2, potentially indicating additional genotypic variation. However, as these sequences were obtained from NCBI, validation of these substitutions using the original trace files was not possible.

## DISCUSSION

Mucormycetous fungi show minimal intrinsic pathogenicity to normal individuals, but they can initiate aggressive and fulminant infections under certain clinical conditions, such as after solid-organ transplantations. It has been shown by multivariate analysis that previous exposure to voriconazole is a significant risk factor for mucormycosis.^19^ Other factors likely associated with an increased risk of mucormycosis include the severity and type of immunosuppression.^20^ Disseminated mucormycosis is an extremely rare and fatal form of the disease.^21, 22, 23^ Despite increasing reports of mucormycosis in recent years^4, 24^ the disease remains uncommon.^1^ It occurs in 0.4%−26.0% of solid-organ transplantation recipients and results in high mortality rates, with the highest incidence among liver transplant recipients (26%–55%).^1, 4, 19, 25, 26^ Mucormycosis in liver transplantation recipients is an extremely rare but life-threatening fungal infection that is frequently associated with impaired immune status, and this population has the worst outcome among solid-organ transplant recipients.^27^ Generally, mucormycosis has a very poor prognosis.

We retrospectively reviewed all human infections caused by the rare emerging fungus *M. indicus* for the period January 1975−December 2016 (Table 2). Of the 13 cases reported in the literature (including the present case), six cases (46.1%) involved the gastrointestinal tract, four cases (30.7%) involved hematologic malignancies and eight (76.9%) patients had disseminated infections, and the mortality rate of patients with *M. indicus* infections was 4/13 (30.7%). Our case is the first pulmonary infection by *M. indicus* in a liver transplant recipient, which disseminated to involve the skin.

Diagnosis remains challenging, as the clinical and radiological presentation can be non-specific and can mimic other fungal infections. Pulmonary mucormycosis progresses and disseminates rapidly. Therefore, it is essential for the clinician to maintain a high level of suspicion, as early diagnosis and aggressive treatment can result in a more favorable outcome. The time between the onset of symptoms and diagnostic procedures has been shown to be associated with the likelihood of mortality.^28^ The most likely portal of entry in the present patient was the lung. CT features of pulmonary mucormycosis in solid-organ transplant recipients include consolidation or mass-like lesions, nodules, or cavities in approximately 25% of patients.^29^ Additionally, mucormycosis remains an uncommon disease with limited options for treatment with which clinicians have only limited experience. The ESCMID and ECCM guidelines for treatment of mucormycosis include antifungal therapy with amphotericin B as a first-line agent^30^ and surgical intervention in most cases with reducing predisposing factors.^31^ Amphotericin B showed a low *in vitro* minimum inhibitory concentration (0.03 μg/mL) value against *M. indicus* with a successful clinical response. These findings were supported by the data of other investigators who reported low *in vitro* minimum inhibitory concentration values of amphotericin B against most mucormycetes.^32, 33^ Despite profound immunosuppression and infection with *M. indicus,* the present patient made a full recovery, which may in part be the result of early diagnosis, treatment, and aggressive surgical intervention. Thoracotomy and removal of the fungal ball was done 2 months after the initiation of antifungal therapy.

In this study, we analyzed a large collection of *M. indicus* isolates, mainly sampled from different clinical and environmental specimens collected worldwide that showed two different genotypes, 1 and 2; the latter was divided into three subtypes, 2a, 2b and 2c. For the D1/D2 region of the *LSU rDNA* gene, the two genotypes differ by only one nucleotide substitution (A−G), whereas the *ITS* sequences yielded four differences between the two genotypes. Based on the sequence type and origin of the strains, we cannot see a correlation between the two based on the available data.

In conclusion, two different *M. indicus* genotypes were observed. Overall, further physiological and morphological studies are needed to assess a possible link between the genotypes and possible virulence factors. *M. indicus* has emerged as a significant cause of invasive mycoses in severely immunocompromised patients, causing serious human infections worldwide. *M. indicus* can infect a wide variety of patients with no real predilection in the site of infection. Early diagnosis and initiation of appropriate therapy may enhance survival in these immunocompromised patient populations.

## Figures and Tables

**Table 1:** Source and GenBank accession numbers of CBS and FMR reference strains of *Mucor indicus* and the strains isolated from Qatar

**No.**	**Strain code**	**Accession LSU**	**Accession ITS**	**Species**	**Genotype**	**Source**	**Country**	**Isolation/deposite year**
1	CBS226.29	FN650669	HM999956	*M. indicus*	2	Unknown	Switzerland	1929
2	FMR 9922	**KT359362**	**KT359357**	*M. indicus*	2	Peritoneal fluid	USA	–
3	FMR 9947	**KT359363**	**KT359358**	*M.indicus*	2	Liver abscess	USA	–
4	FMR 9996	**KT359364**	**KT359359**	*M. indicus*	2	Liver abscess	USA	–
5	FJ M 55	KM527248	–	*M. indicus*	2	Distiller’s yeast	China	–
6	L7	KM527240	–	*M. indicus*	2	Distiller’s yeast	China	–
7	CNRMA03 894	–	DQ118993	*M. indicus*	2	Human, stomach	Germany	–
8	UTHSC02 2453	–	FN663956	*M. indicus*	2	Hand wound	Rhode Island, USA	–
9	CBS 671.79	–	JN206183	*M. indicus*	2	Fermentation of rice-tape; Fermentation of cassava-tape	Jakarta, Indonesia	1979
10	CBS 120.08	–	JN206182	*M. indicus*	2	Unknown	Unknown	1908
11	CBS 123974	–	JN206181	*M. indicus*	2	Human; gastrointestinal infection	Germany	2008
12	CBS 120585	–	JN206180	*M. indicus*	2	Human; muscle	India	2006
13	F5 02	–	JN561265	*M. indicus*	2	Isolated from wine starter samples	China	
14	NN2703	–	EU798706	*M. indicus*	2	Loogpang/amylolytic starter (production process Sato, traditional Thai rice wine)	Thailand	–
15	ss6	–	JN974014	*M. indicus*	2b	Microbiology laboratory on board Sagar Sampada	India	–
16	UTHSC01 667	–	FN663955	*M.indicus*	2a	Stoma tissue	Pennsylvania, USA	–
17	1607001088 (ATCC MYA 4678)	**KT359360**	**KT359355**	*M. indicus*	1	BAL specimen from patient from a disseminated mucormycosis infection	Qatar	2008
18	16071106	**KT359361**	**KT359356**	*M. indicus*	1	Wound/skin from patient from a disseminated mucormycosis infection	Qatar	2008
19	CBS 535.80	–	JN206184	*M. indicus*	1	Sorghum malt	South Africa	1980
20	CBS 414.77	–	JN206185	*M.indicus*	1	Dung of berber goat	India	1977
21	CBS 422.71	–	JN206186	*M.indicus*	1	Dioscorea; tuber	Indonesia	1971
22	SUMS0707	–	KF752482	*M. indicus*	1	Ulcer secretion, necrotizing fasciitis	China	–
23	CBS 837.70	JN206508	JN206175	*M. variisporus*	–	Unknown	India	1970
24	CBS 763.74	HM849688	HM999957	*M. amphibiorum*	–	Amphibian	Germany	1974
25	CBS 255.36	JN206502	JN206177	*M.inaequisporus*	–	Spondias mombin; fruit	Ghana	1936
26	CBS 674.88	JN206507	–	*M.ucrainicus*	–	Soil of litter layer	Germany	1988
27	CBS 221.71	–	JN206191	*M.ucrainicus*	–	Dung of mouse	Ukraine	1971
28	CBS 210.80	JN206504	JN206172	*M.ardhlaengiktus*	–	Garden soil	India	1980
29	UTHSC02 2090	FN650660	FN650647	*M.ellipsoideus*	–	Peritoneal dialysis fluid	Florida, USA	–

Accession numbers in bold are of sequences created in this study.

**Table 2:** Cases of *Mucor indicus* mucormycosis reported in the literature^a^

**Case no.**	**Year of infection**	**Gender/Age (years)**	**Underlying disease**	**Site of infection**	**Dissemination**	**Histopathology/direct microscopy**	**Treatment**	**Outcome**	**Reference**
1	1975	66/M	None	Epigastric mass	No	+	AmB (0.4 mg/kg/d, 4 times/wk; 2 mo (Partial gastrectomy)	Survival	Douvin *et al.*^34^
2	1985	Infant (not specified)	Premature	Pulmonary	Yes	+	None	Died	Krasinski, K.^b^,^21^
3	1990	F/27	T-lymphoblastic leukemia, chemotherapy-induced neutropenia	Ileocaecal region, liver	Yes	(+) on direct examination	AmB+5-FC (doses unknown; 5 d)	Died	Ter Borg, F.^22^
4	1996	M/39	Bone marrow transplant recipient, prednisone treatment of graft-versus-host disease	Liver	No	(+) on direct examination	AmB (1.0 mg/kg/d;14 d. switched to ABLC (5.0 mg/kg; duration unknown (percutaneous drainage of liver abscesses)	Survived	Oliver, M.R.^35^
5	2001	F/82	Apparently healthy	Fasciitis, muscles, bone on the left knee, with fever	Yes	+	AmB (dose and duration unknown)+amputation	Survived	Mata-Essayag, S.^36^
6	2001	F/56	None	Discomfort in the vulva, itching, and stinging sensation with vaginal discharge	No	(+) on direct examination	Topical AmB (3% cream), 5 g/d; 28d, and on alternate days for 2 mo	Cure	Sobel, J.D.^37^
7	2006	M/34	None	Colon, fungemia	Yes	(+) on direct examination	ABLC (5 mg/kg/d), later LAmB (5 mg/kg/d), for 6 wk	Survived	Aboltins, C. A.^38^
8	2006	M/48	Acute head injury	Stomach, ileocaecal valve, colon	Yes	+	LAmB (7.5 mg/kg/d;33d) Hemicolectomy	Survived	Deja M.^39^
9	2008	F/6 mo	Berlin LVAD heart implant	Left and right ascending aorta, pericardium	Yes	(+) on direct examination	Sterleedingnotomy, uncontrollable b	Died	De Repentigny, L.^23^
10	2010	F/47	AML, neutropenic fever on chemotherapy	Interdental periodontal aspects of the left second premolar and first molar teeth	No	+	LAmB (5 mg/kg/d), micafungin (mg/kg/d), and oral rinse AmB, for 4wk	Survived	McDermott, N.E.^40^
11	2013	F/62	ALL, HLA- haploidentical peripheral blood stem cell transplantation	Liver and sigmoid colon	Yes	?	LAmB (dose?)	Died	Koteda^c^,^8^
12	2014	M/58	None	Ulceration at right peritibial region are due to injury by a brick	No	+	AmB (50 mg/d) a total of 2126 mg (47 d), then oral itraconazole (200 mg/d) for 3 mo. Skin grafting	Survival	Luo, Y.^41^
13	This case	M/55	Liver transplant on immunosuppressive therapy	Lung, skin	Yes	+	LAmB (250 mg/for 3 mo, right anterolateral 5th intercostal thoracotomy with removal of fungal ball	Survival	Present study

Abbreviations: amphotericin B lipid complex, ABLC; liposomal amphotericin B, LAmB; amphotericin B, AmB; acute myeloid leukemia, AML; acute lymphoblastic leukemia, ALL.

a Data were recorded by carefully scrutinized references for single case reports. Then this initial review was expanded by a MEDLINE and Google Scholar using the key word: *Mucor indicus*.

b Cited as *Rhizopus indicus*.

c English abstract of article in Japanese.
